# Supplementary material for: Retrospective Analysis of the Correlation of MSI-h/dMMR Status and Response to Therapy for Endometrial Cancer: RAME Study, a Multicenter Experience
Source: Cancers (Basel). 2023 Jul 15;15(14):3639. doi: 10.3390/cancers15143639 (PMC10377998; doi:10.3390/cancers15143639)
Supplement: Supplementary file 1 [file cancers-15-03639-s001.zip › cancers-2471531-supplementary.pdf]

Table S1. Patients with dMMR (N=63) diagnosed with IHC, details of MMR proteins.
N = numer; MMR = mismatch repair; IHC = immoistochemistry

N= 63	Normal	Absent
MMR proteins (IHC)		
MLH 1	13	50
PMS 2	15	48
MSH 2	51	12
MSH 6	51	12
Details of N. of absent MMR protein at IHC		
4 absent proteins		4/63
3 absent proteins		0/63
2 absent proteins		47/63
MLH1 + PMS2		40/63
MLH1 + MSH2		2/63
MSH2 + MSH6		5/63
1 absent protein		
MLH 1		4/63
PMS 2		4/63
MSH 2		1/63
MSH 6		3/63
